# Supplementary material for: Nuclear envelope budding is a response to cellular stress
Source: Proc Natl Acad Sci U S A. 2021 Jul 21;118(30):e2020997118. doi: 10.1073/pnas.2020997118 (PMC8325156; doi:10.1073/pnas.2020997118)
Supplement: Supplementary File [file pnas.2020997118.sapp.pdf]

PNAS
www.pnas.org

Supplementary Information for
Nuclear envelope budding is a response to cellular stress

Dimitra Panagaki[a1] and Jacob T. Croft[a1], Katharina Keuenhof[a], Lisa Larsson-Berglund[a], Stefanie Andersson[a], Verena Kohler[c], Sabrina Büttner[c], Markus J. Tamás[a], Thomas Nyström[b], Richard Neutze[a], Johanna L. Höög[a*]

*Johanna L. Höög
Email: johanna.hoog@gu.se

This PDF file includes:

Supplementary text
Figures S1 to S7
Tables S1 to S6
SI References

[1] These authors contributed equally to the manuscript.

Supplementary figure 1 (Related to Figure 5)

HMC-1 cells (unstressed)

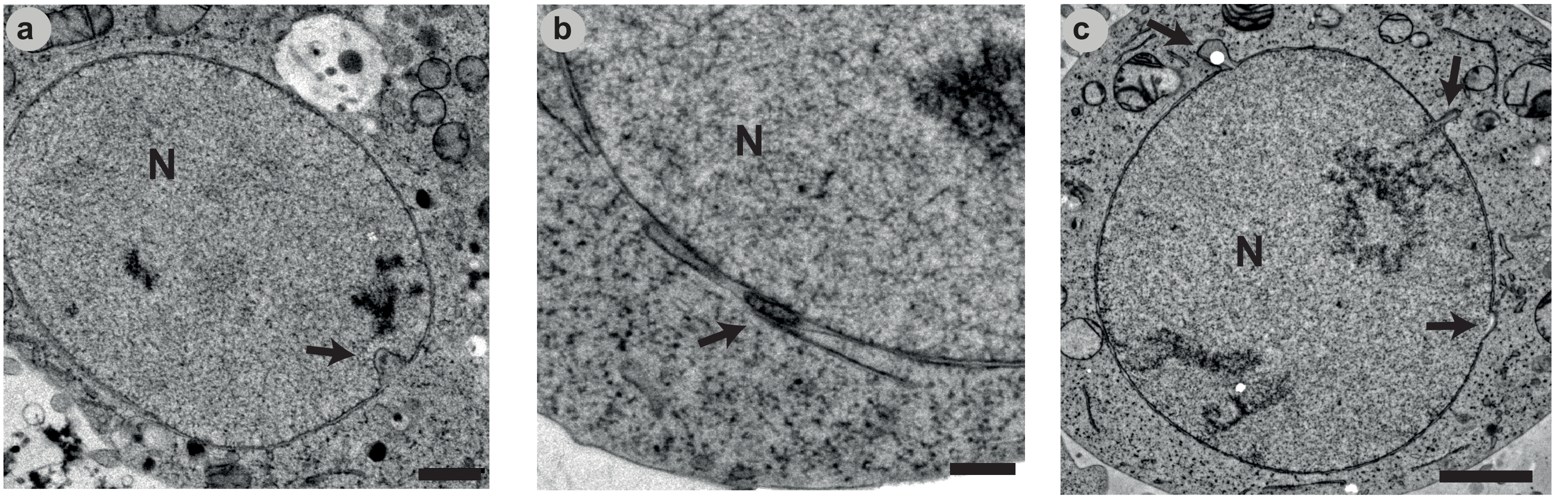

Saccharomyces cerevisiae (unstressed cells)

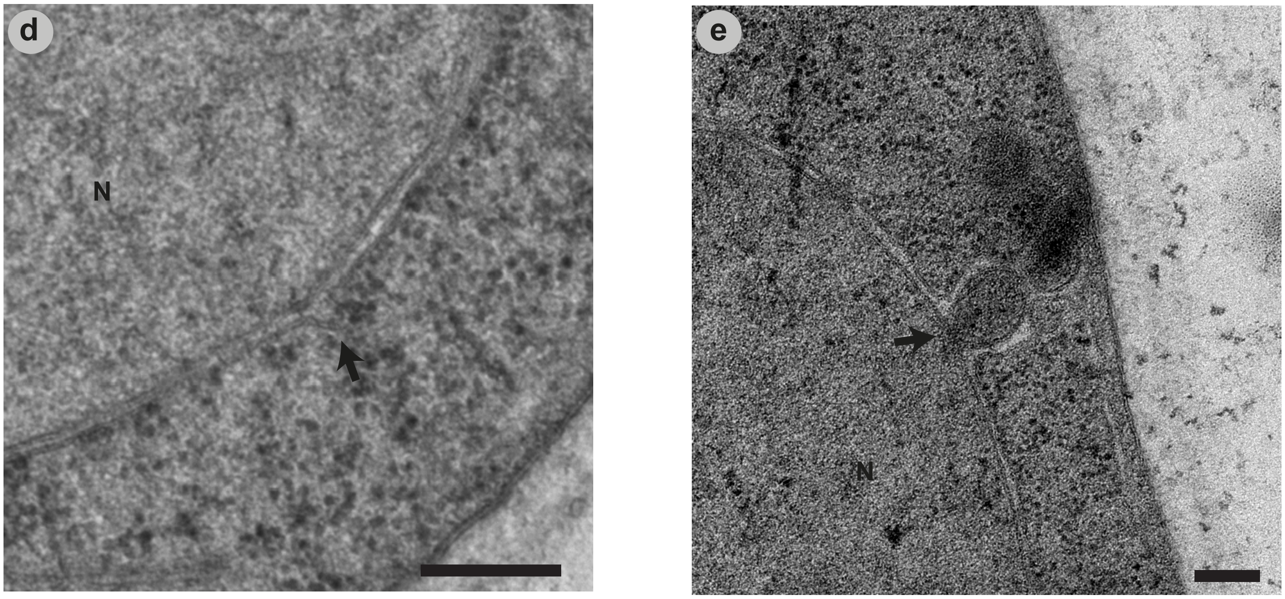

Saccharomyces cerevisiae (stressed cells)

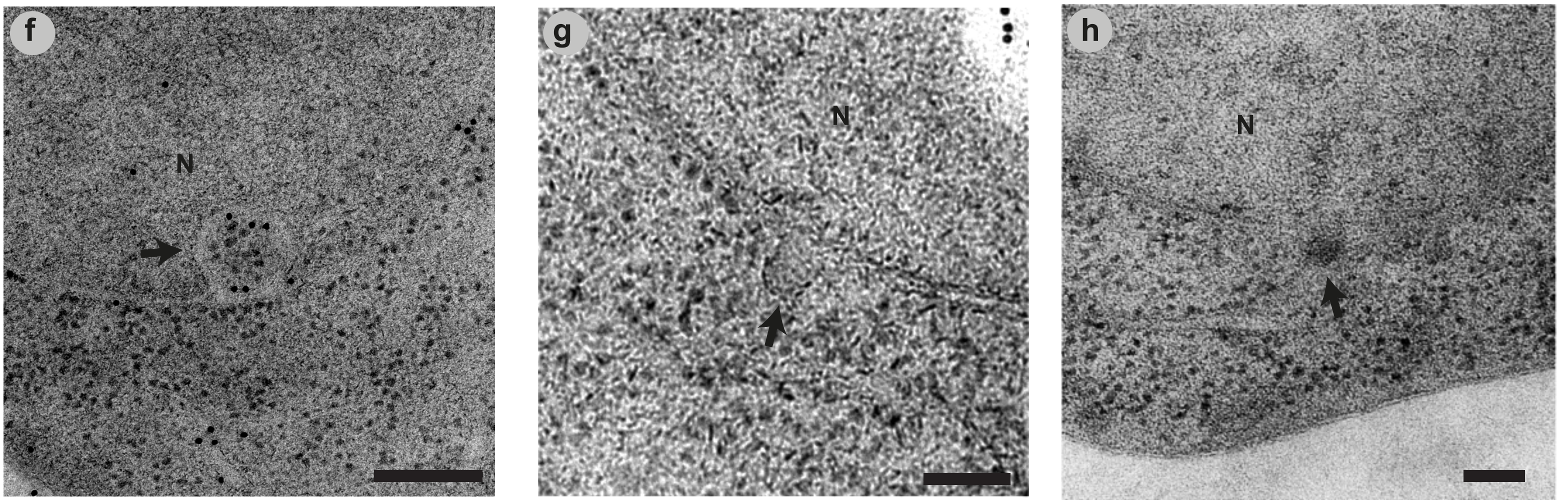

Supplementary figure 1. Different morphologies of NEB events. Electron microscopy of thin sections revealed several NEB events in (**a-c**) HMC-1 cells and (**d-h**) *S. cerevisiae* with unique morphologies. These could correspond to different stages in the same process, or a variety of processes that occur by NEB. (**a**) NEB in which the event protruded towards the nucleoplasm (Type 2 NEB). (**b**) NEB in which a vesicle was clearly inside the perinuclear space but with no distinct directionality (Type 3 NEB). (**c**) Two NEB events occurring in the same nuclear section. (**d**) NEB event showing only a small protrusion of the outer nuclear membrane with no material apparent inside. (**e**) Outwards protruding NEB event containing two vesicles instead of one. One vesicle appears complete however the second is still continuous with the inner nuclear membrane. (**f**) Inwards protruding NEB observed in a yeast culture that was subjected to heat shock. The event contains a complete vesicle within, and the contents have similar electron density to cytoplasm with ribosomes clearly visible. (**g**) NEB event showing only a small protrusion of the outer nuclear membrane and no material apparent inside. This event was observed in an aged cell population. (**h**) Electron dense particle similar in appearance to the cargo of NEB observed in connection with the ER. This event was observed in a cell exposed to oxidative stress. Scale bars: 2 µm (**a, c**), 1 µm (**b, d**), 200 nm (**e-h**). Abbreviations: N, nucleus; NEB, nuclear envelope budding; HMC-1, human mast cell line 1; black arrows indicate NEB events.

Supplementary figure 2 (Related to Figure 1)

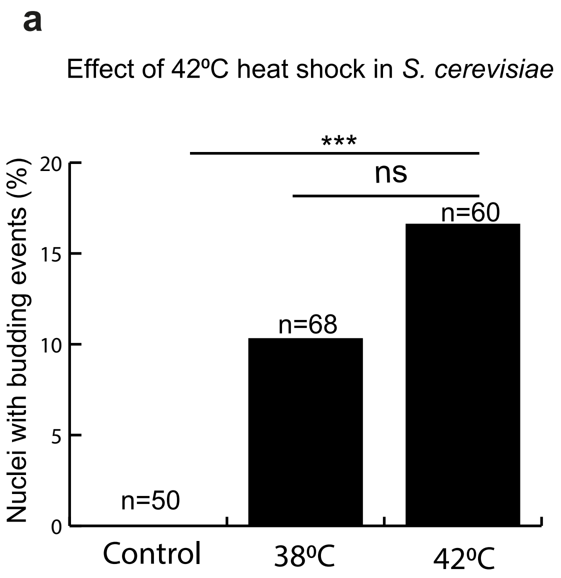

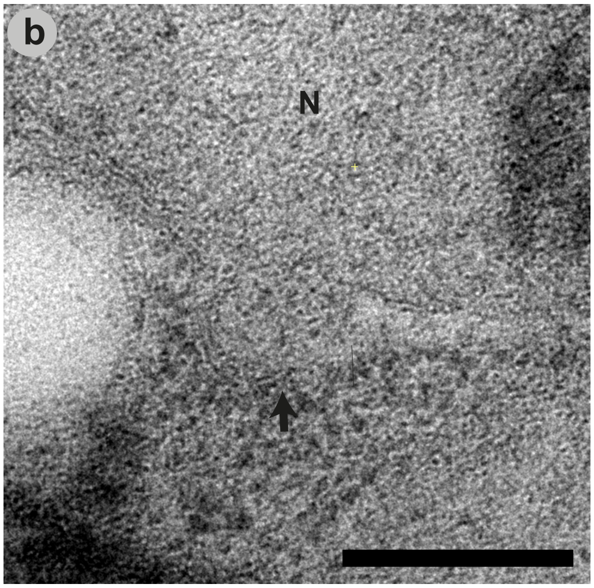

Supplementary figure 2. Effect of 42ºC heat shock in *S. cerevisiae*. Cells were subjected to 42ºC heat shock for 30 minutes to evaluate the influence of a higher temperature in the frequency of NEB. (**a**) The 42ºC heat shock increased the frequency of NEB to a higher extend compared with control cells. (**b**) Representative micrograph of a NEB event found under the 42ºC heat shock treatment. ***P<.001, ns no significant differences between groups. Scale bar: 200nm.

Supplementary figure 3 (Related to Figure 2)

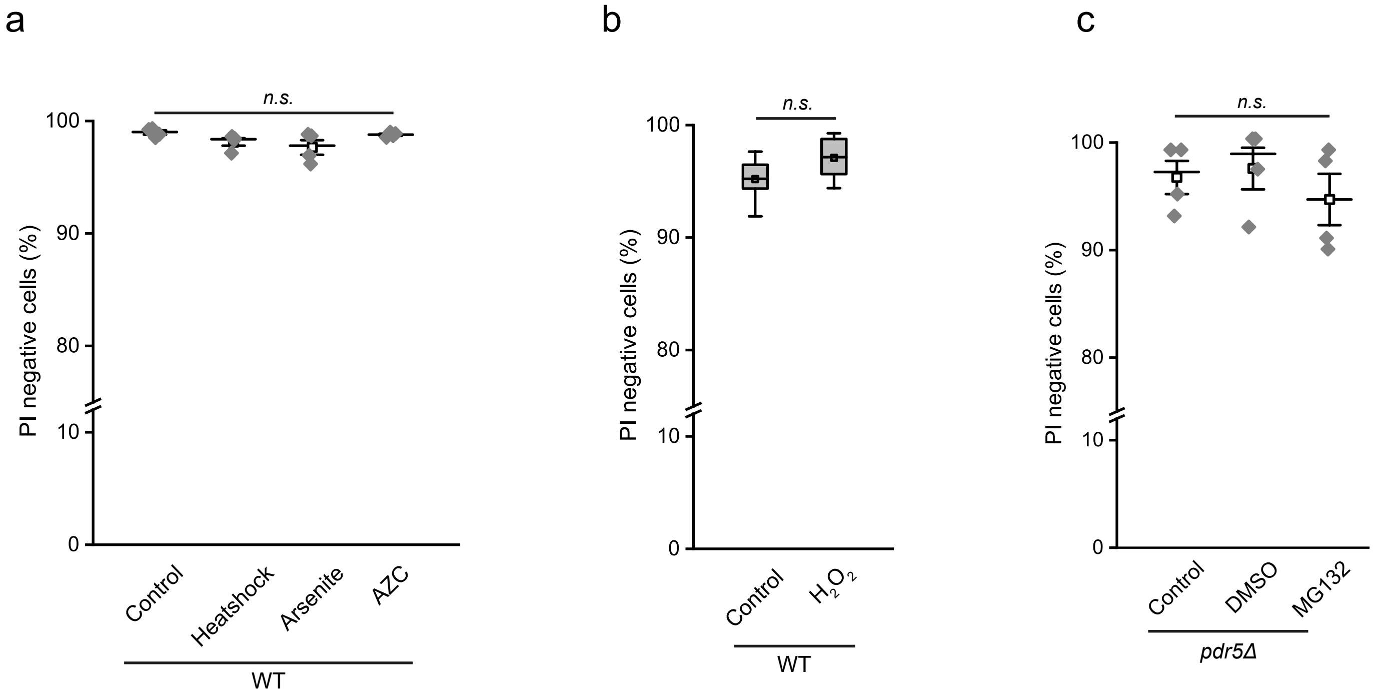

Supplementary figure 3. Viability assay of cellular stressors. For evaluating the condition of the cells after subjected to variant stressors, propidium iodide (PI) staining was performed. (**a-c**) Percentage of PI negative cells for each stressor and reagent that may have affected the viability of the cells. There was no significant difference between the control and the treated groups as illustrated in the graphs.

Supplementary figure 4 (Related to Figure 3)

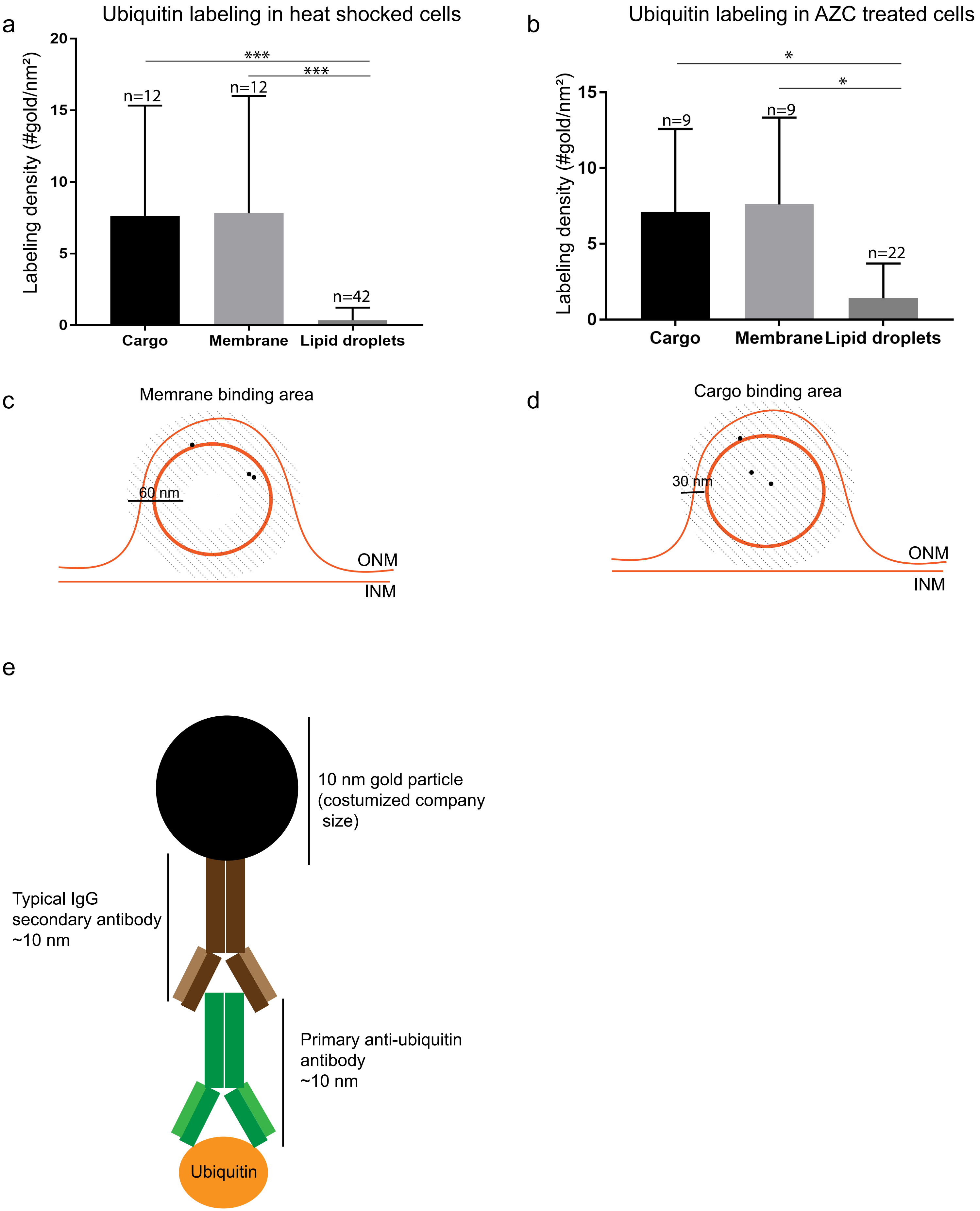

Supplementary figure 4. Localization of immuno-labeling gold particles. The localization of the gold particles for the ubiquitin assay, was characterized as ‘membrane binding’ or ‘cargo binding’ in accordance with their distance from the respective structures. (**a, b**) Labeling density of the ‘cargo binding area’, the ‘membrane binding area’ and lipid droplets (negative control). (**c, d**) Definition of the ‘cargo binding area’ and the ‘membrane binding area’. As the antibody sandwich that was used for this assay has a length of approximately 30 nm, the total area of the cargo including 30 nm of the surrounding area, was considered as the ‘cargo binding area’. In a similar manner, the area stretching 30 nm inwards and outwards of the cargo’s membrane was considered as the ‘membrane binding area’. (**e**) A graphical representation of the antibody sandwich and its approximate length. All graphs were generated using GraphPad Prism (version 8.1.2 (332)) software with the error bars representing the standard deviation. *$P<.05$, ****$P<.0001$ vs. lipid droplets (negative control).

Supplementary figure 5 (Related to Figure 6)

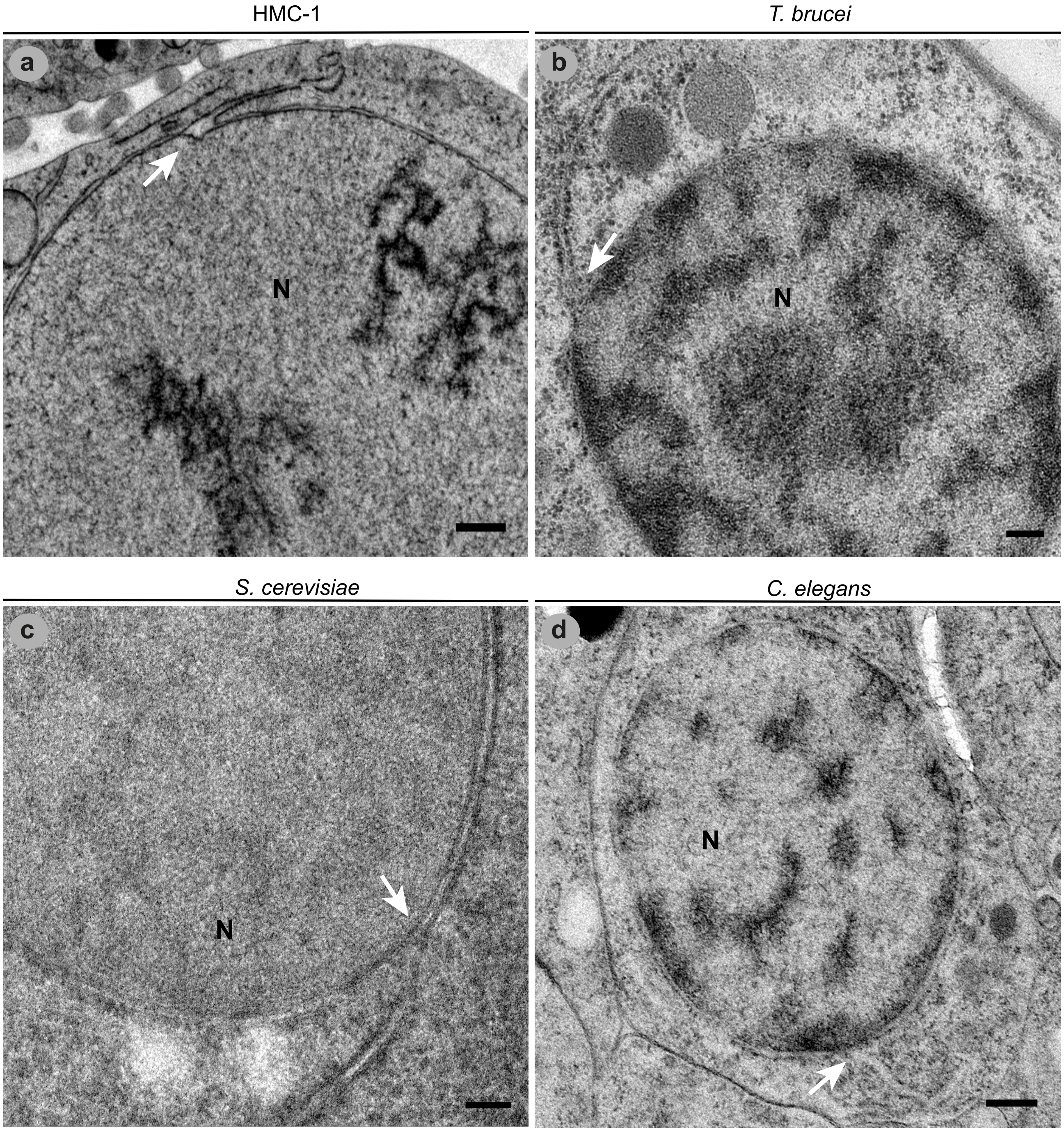

Supplementary figure 5. Examples of NE-ER connections. (**a**-**d**) NE-ER connections as they appear in 2D electron microscopy pictures in four different organisms. Their morphology is profoundly different from the one of the NEB events described in this study. Scale bars: 2 µm (**a**), 300 nm (**b**), 200 nm (**c**), and 500 nm (**d**). Abbreviations: NE, nuclear envelope; ER, endoplasmic reticulum; NEB, nuclear envelope budding; N, nucleus; white arrows indicate the NE-ER connection points.

Supplementary figure 6 (Related to Figure 5)

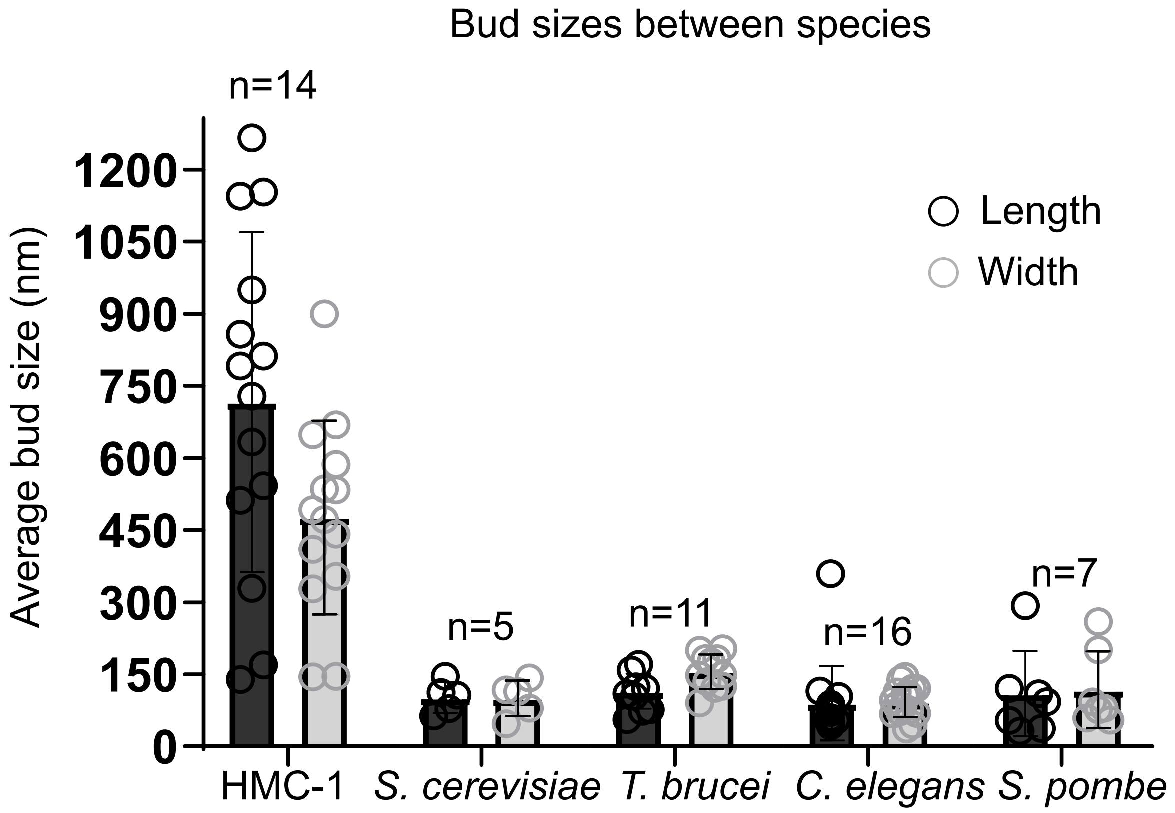

Supplementary figure 6. Different sizes of NEB events. Average sizes of nuclear buds (length and width) for each species. The n is equal to the total number of buds for the respective organism. Abbreviations: NEB, Nuclear envelope budding; HMC-1, Human mast cell line 1. The graph was generated using the GraphPad Prism (version 8.1.2 (332)) software with the error bars representing the standard deviation.

Supplementary figure 7 (Related to Figure 3)

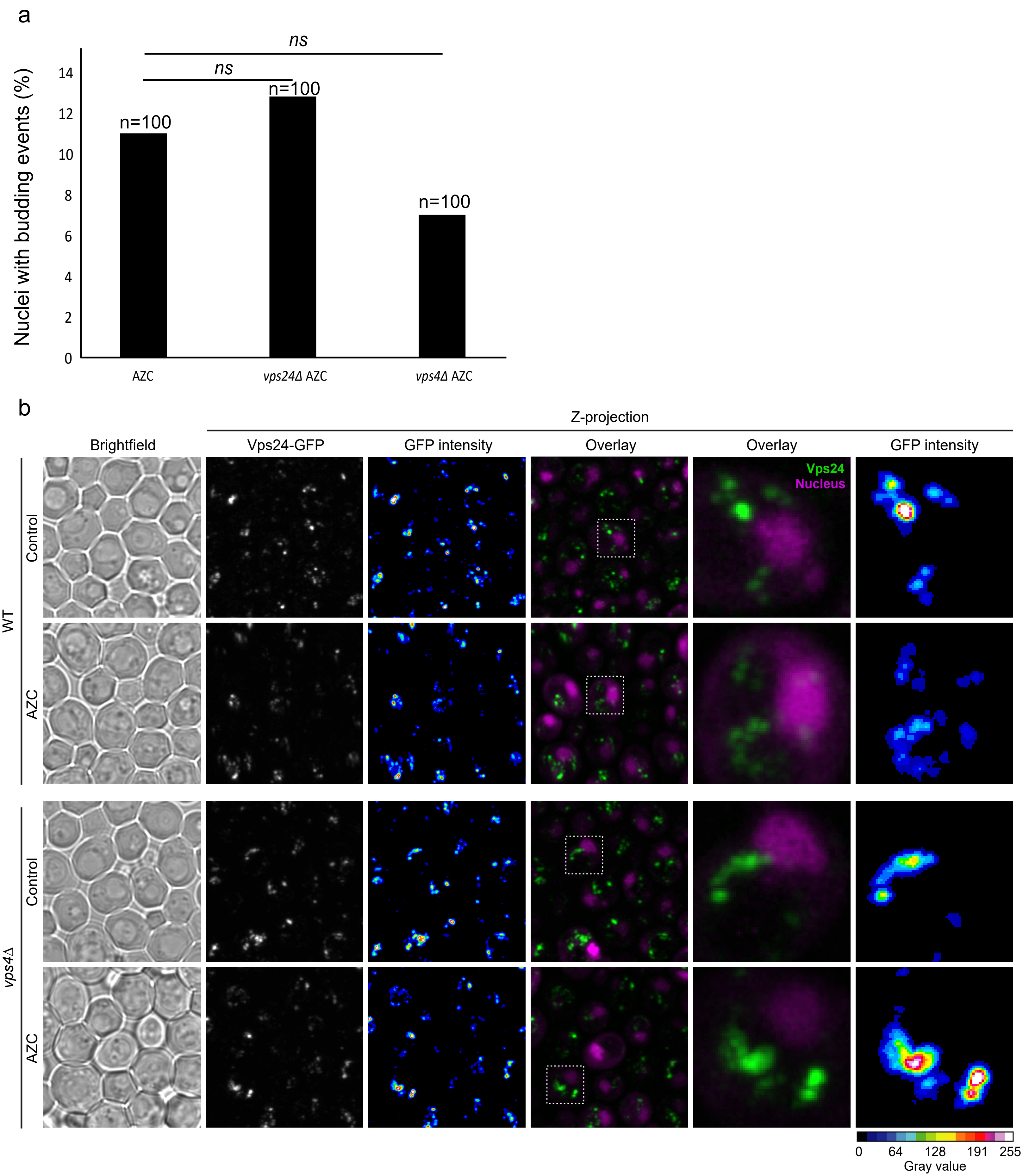

Supplementary figure 7. No evidence for involvement of the ESCRT pathway in NEB. (**a**) The possible involvement of the ESCRT pathway in the formation of NEB events, was examined through deletions of *VPS24* and *VPS4* genes. NEB frequency in the two deletion strains, *vps24Δ* and *vps4Δ* showed no significant difference to the control. In this experiment, all samples were treated with AZC for 90 minutes to enable the detection of a potential decrease in NEB frequency. (**b**) Confocal microscopy images of the Vps24-GFP protein both in wild type and *vps4Δ* strains show that the ESCRT protein labelled did not colocalize with the nucleus counterstained with DRAQ5. These experiments were performed both in normal and AZC treated conditions. Scale bars: 2μm.

Supplementary Table 1

Supplementary table 1. List of organisms where NEB events have been previously observed, presented in a chronological order. The different terminologies used to describe the events are also included. *In this organism, the morphology of the budding events was somewhat different from the rest.

Organism	Cell type	Year of publication	Terminology	Reference
Drosophila melanogaster	Salivary gland cells	1955	Membrane outpocketings	(Gay, 1955)
Oryctolagus cuniculus	Blastocysts	1962	Nuclear extrusion	(Hadek et al., 1962)
**Noctiluca scintillans*	unicellular	1963	Annulated vesicles	(Afzelius, 1963)
Cricetulus griseus	Embryo cell line A_1	1965	Nuclear budding	(Longwell et al., 1965)
Rattus norvegicus	Fertilized oocytes	1965	Nucleolar extrusion	(D. Szollosi, 1965)
Bos taurus	Fibroblastic cells	1965	Nuclear buds	(Elston et al., 1965)
Tradescantia bracteata	Developing microspores	1969	Membrane bounded bodies	(Mepham et al., 1970)
Podocarpus macrophyllus	Haploid microspore tetrads	1969	Invaginations of nuclear envelope	(Aldrich et al., 1970)
Lycopodium annotinum L.	Immature spores	1970	Extrusions/bulges	(Gullvåg, 1970)
Pinus banksiana	Post meiotic microspores	1970	Folding of nuclear envelope	(Dickinson et al., 1970)
Lilium longiflorum	Young microspores	1971	Membrane-bound inclusions	(Dickinson, 1971)
Drosophila melanogaster	-Salivary gland cells<br>-midgut cells	1987	Pod-like infoldings of the nuclear envelope	(Hochstrasser et al., 1987)
Mus musculus	-Zygotes<br>-early embryos<br>-hybrid cells	1988	‘Blebbing’ of nuclear envelope	(M. S. Szollosi et al., 1988)
Drosophila melanogaster	Larval muscle cells	2012	Nuclear envelope budding	(Speese et al., 2012)
Strongylocentrotus purpuratus	Embryonic cells	2018	Nuclear egress	(LaMassa et al., 2018)
Saccharomyces cerevisiae	Aging mitotic cells/NPC assembly mutants	2019	Herniations at the nuclear envelope	(Rempel et al., 2019)
Drosophila melanogaster	Salivary gland cells	2020	Nuclear envelope budding	(Verboon et al., 2020)

**Supplementary Table S2:** Summary of experimental observations							
	Measurements (N)	Observations (x)	x/N (%)	*p*-values are calculated against indicated control data ‡Wilcoxon	$Fisher	£chi-squared	€t-test
**Figure 1a:** Effect of heat shock in *S. cerevisiae*							
**EDC**							
Control wild type cells	76	27	35.5%				
5 min	63	19	30.2%	0.5064	0.8023	0.5032	0.5067
15 min	72	60	83.3%	$< 10^{-4}$	$< 10^{-4}$	$< 10^{-4}$	$< 10^{-4}$
30 min	68	55	80.9%	$< 10^{-4}$	$< 10^{-4}$	$< 10^{-4}$	$< 10^{-4}$
45 min	70	57	81.4%	$< 10^{-4}$	$< 10^{-4}$	$< 10^{-4}$	$< 10^{-4}$
90 min	81	49	60.5%	0.0018	0.0014	0.0018	0.0016
**NEB**							
Control wild type cells	337	8	2.4%				
5 min	63	3	4.8%	0.289	0.2426	0.2874	0.2886
15 min	72	4	5.6%	0.1475	0.1429	0.1465	0.1472
30 min	68	7	10.3%	0.0016	0.0059	0.0016	0.0016
45 min	70	4	5.7%	0.1337	0.1337	0.1327	0.1334
90 min	81	4	4.9%	0.2158	0.1864	0.2146	0.2156
15 min + 30 min + 45 min	210	15	7.1%	0.0069	0.0072	0.0069	0.0068
**Figure 2d:** Effect of cell stress on NEB frequency							
**NEB events**							
Control wild type cells	337	8	2.4%				
Arsenite treated	249	15	6.0%	0.0247	0.0215	0.0245	0.0245
Young cells	204	14	6.9%	0.0105	0.0107	0.0104	0.0104
Old cells	200	18	9.0%	0.0006	0.0007	0.0005	0.0005
Old cells vs. Young cells				0.4275	0.2708	0.4264	0.4277
*H_2O_2 control	197	3	1.5%	0.5052	0.8369	0.5041	0.505
*H_2O_2 treated	204	12	5.9%	0.0363	0.0332	0.0361	0.0361
*H_2O_2 treated vs. H_2O_2 control				0.0217	0.0189	0.0215	0.0214
**Figure 2f:** Effect of AZC on NEB frequency							
**NEB events**							
Control wild type cells	337	8	2.4%				
AZC treated cells 30 min	114	3	2.6%	0.8788	0.5552	0.8775	0.8778
†AZC treated cells 90 min	100	22	22.0%	$< 10^{-4}$	$< 10^{-4}$	$< 10^{-4}$	$< 10^{-4}$
**Figure 3c:** Proteasome inhibition							
**NEB events**							
Control wild type cells	337	8	2.4%				
rpn4Δ	100	15	15.0%	0.0000	0.0000	0.0000	0.0000
pdr5Δ + DMSO	200	5	2.5%	0.5689	0.9268	0.9277	0.9269
pdr5Δ + MG132	200	14	7.0%	0.0094	0.0089	0.009	0.0089
pdr5Δ + MG132 vs. pdr5Δ + DMSO				0.0287	0.0344	0.0347	0.0344
**Figure 4e:** Localization of NPC proteins							
**Immuno-gold labeling events**							
NPC unstressed cells	100	81	81.0%				
NPC aged cells	95	63	66.3%	0.0148	0.0197	0.0201	0.0196
LDs unstressed cells	83	15	18.1%				
LDs aged cells	100	8	8.0%	0.0320	0.0371	0.0378	0.0372
LDs vs NPCs unstressed cells				$< 10^{-4}$	$< 10^{-4}$	$< 10^{-4}$	$< 10^{-4}$
LDs vs NPCs aged				$< 10^{-4}$	$< 10^{-4}$	$< 10^{-4}$	$< 10^{-4}$
NEBs unstressed cells	6	1	16.7%				
NEBs aged cells	10	1	10.0%	0.6250	0.6963	1.0000	0.7192
NEBs vs. NPCs unstressed cells				0.0238	0.0144	0.0152	0.0141
NEBs vs NPCs aged cells				$< 10^{-4}$	$< 10^{-4}$	$< 10^{-4}$	$< 10^{-4}$
**Supplementary Figure 1:** Effect of 42 ^{o}C heat shock in S. cerevisiae							
**NEB events**							
Control wild type cells	337	8	2.4%				
Heatshock 38 °C	68	7	10.3%	0.0016	0.0059	0.0016	0.0016
Heatshock 42 °C	60	10	16.7%	0.0000	0.0001	0.0000	0.0000
Heat shock 42 °C vs. 38 °C				0.2891	0.212	0.2928	0.2928
**Supplementary Figure 4:** Involvement of the ESCRT pathway in NEB							
**NEB events**							
*AZC treated wild-type cells 90 min	100	11	11.0%				
AZC treated *Vps24Δ* cells 90 min	100	13	13.0%	0.6658	0.7427	0.6634	0.6653
AZC treated *Vps4Δ* cells 90 min	100	7	7.0%	0.3254	0.2297	0.323	0.3254

†Experiments involving H_2O_2 treated cells grew on different growth media and therefore an independent control was required.
*These experimental data were not merged to avoid potential batch-by-batch variations in AZC
‡https://se.mathworks.com/help/stats/ranksum.html
$https://se.mathworks.com/help/stats/fishertest.html
£https://se.mathworks.com/matlabcentral/fileexchange/45966-compare-two-proportions-chi-square
€https://se.mathworks.com/help/stats/ttest2.html

Supplementary table 3

Supplementary table 3. Detailed list and information of all antibodies and reagents used in this study.

Antibodies		
Anti-Nuclear Pore Complex Proteins antibody [Mab414]	Abcam	ab24609
Anti-Ubiquitin antibody (for immuno-EM)	Abcam	ab19247
Anti-GFP antibody	Abcam	ab6556
Anti-Hsp104 antibody	Abcam	ab69549
Rabbit anti-mouse immunoglobulins	Agilent/Dako	E0433
Gold-conjugated protein A	CMC UMC Utrecht	E1808
Goat-anti-Rabbit IgG (H&L), 10nm	Electron Microscopy Sciences	Cat#25108
Anti-Ubiquitin antibody (for WB)	Santa cruz	Sc-8017
Chemicals		
Uranyl acetate	SPI-CHEM	Lot#1221013
HM20 non-polar Lowicryl	Polysciences Europe GmbH	Cat#15924-1
Lead Nitrate	Merck	Cat#109969
Sodium Citrate	Merck	Cat#111037
Cationic gold particles (15 nm)	British Bio Cell	SKU#: EM.GC15
Osmium tetroxide	TAAB	Cat#O014
Fish skin gelatin	Sigma-Aldrich	Cat# 9000-70-8
Sulfo-NHS-LC biotin	Thermo Fisher scientific	Cat#21335
Streptavidin magnetic beads	Thermo Fisher scientific	Cat#21344
Glutaraldehyde	Sigma-Aldrich Sweden AB	Cat#3802
Sodium arsenite	Sigma-Aldrich	Cat# S7400
1-Hexadecene	Merck	Cat#629-73-2
L-Azetidine-2-carboxylic acid (AZC)	Bachem	Cat# F-1281
Hydrogen peroxide	Sigma-Aldrich	Cat# 7722-84-1
Propidium iodide	Sigma-Aldrich	Cat#81845
DRAQ5	Abcam	Ab108410
MG132	Sigma-Aldrich	Cat#C2211

Supplementary table 4

Supplementary table 4. Detailed list and information of all yeast strains and primers used in this study.

Yeast strains

Strain	Genotype	Source
BY4741	MATa, *his3*Δ1, *leu2*Δ0, *met15*Δ0, *ura3*Δ0	Euroscarf
BY4741 *vps4*Δ	BY4741 *vps4*Δ::hphNT1	This study
BY4741 *vps4*Δ Vps24^{GFP}	BY4741 *vps4*Δ::hphNT1 *VPS24*-yeGFP-kanMX	This study
BY4741 Vps24^{GFP}	BY4741 *VPS24*-yeGFP-kanMX	This study
BY4741 *vps24*Δ	BY4741 *vps4*Δ::hphNT1	This study
BY4741 *pdr5*Δ	BY4741 *pdr5*Δ::hphNT1	This study

Oligonucleotides

Modification	Oligonucleotides	PCR template
Deletion of *VPS4*	5’-ATGGAAGACAAAAATAAAGCAGCATAGAGTGCCTATAGTAGATGGG GTACAAATGCGTACGCTGCAGGTCGAC -3’<br>5’-TTTTTTATTTTCATGTACACAAGAAATCTACATTAGCACGTTAATCAA TTGACTAATCGATGAATTCGAGCTCG-3’	pFA6a-hphNT1
Control PCR *VPS4* deletion	5‘-GTCGACCTGCAGCGTACG-3<br>5’- GATTCACATGTCGCCACTCCAGTC-3’	
C-terminal tagging of *VPS24*	5’-CATTATTTATTCACTTATTTATTTATTTTCTTTGTACAGTCACAGTAA CACTCTAATCGATGAATTCGAGCTCG-3’<br>5’-GGAAGCAGATAGGATGGTAAATGAAATGCGTGAAAG GCTGAGAGCTTTGCAAAACCGTACGCTGCAGGTCGAC-3’	pYM12
Control PCR *VPS24* tagging	5‘-CGAGCTCGAATTCATCGAT-3‘<br>5’-GTGGGATCCAAAGACTGGAAC-3’	
Deletion of *VPS24*	5’-ACCTTTAGTAGTTTGGGGGGCAGTTTTCTGGGCAATACAAAGTTTA CTTTTGATGCGTACGCTGCAGGTCGAC -3’<br>5’-CATTATTTATTCACTTATTTATTTATTTTCTTTGTACAGTCACAG	pFA6a-hphNT1

Supplementary table 5

Supplementary table 5. Summarized protocols on thin sections and tomography and immuno-EM samples.

**Species**	**High-pressure freezing**	**Freeze substitution**	**Embedded plastic**	**Imaging**
S. pombe	Leica EM PACT1	Long protocol (UA, GA, OsO_4)	HM20	FEI Tecnai TF20
T. brucei	Leica EM PACT2	Short protocol (UA)	HM20	Tecnai T12/Tecnai TF30 300 kV IVEM
S. cerevisiae	Wohlwend Compact 3	Short protocol (UA)	HM20	Tecnai T12/Tecnai TF30 300 kV IVEM
C. elegans	Wohlwend Compact 3	Modified short protocol (UA)	HM20	Tecnai T12
HMC-1	Leica EM PACT1	Short protocol (UA)	K4M	Leo 912AB Omega TEM 120 kV
**Antibody**	**Dilution**	**Incubation time**	**Experiment**	**Order**
mAb414	1:50	2 hours	NPC labeling	First incubation
Rabbit anti-mouse immunoglobulins	1:150	1 hour	NPC labeling	Second incubation
10nm gold-conjugated protein A	1:70	30 minutes	NPC labeling	Third incubation
Ab19247	1:20	2 hours	Ubiquitin labeling	First incubation
10nm gold Goat-anti-Rabbit IgG	1:20	1 hour	Ubiquitin, GFP, Hsp104 labeling	Second incubation
Ab6556	1:5, 1:10, 1:30	Overnight, 4°C	GFP labeling	First incubation
Ab69549	1:100	Overnight, 4°C	Hsp104 labeling	First incubation

Supplementary table 6

Supplementary table 6. Detailed description of the materials and methods used in this project.

**HMC-1 cells**
Human mast cell line 1 (HMC-1; (1)) cells were cultured in Iscove's modified Dulbecco's medium (IMDM). The cells were then treated with 10% exosome-depleted fetal bovine serum (FBS), 100 units/ml streptomycin, 100 units/ml penicillin, 2 mM L-glutamine, and 1.2 U/ml alpha-thioglycerol in incubators kept at 37°C and 5% carbon dioxide (2, 3).
Trypanosoma brucei
Procyclic *T. brucei* strain 427 was cultured in SDM-79 media with 20% fetal bovine serum. Cultures were prepared and maintained at a concentration of 5×10^5 and 1×10^7 cells per mL (4-6).
Schizosaccharomyces pombe
Logarithmically growing wild type fission yeast *S. pombe* were grown at 30°C in YE5S medium (2, 7, 8).
Caenorhabditis elegans
The *C. elegans* wild type reference strain was the Bristol N2 variety. The worms were cultured on normal growing media plates (NGM plates) and the *E. coli* strain OP50 was used as a food source. The worms were maintained at the optimal temperature of 25°C (9, 10). Adult worms were used for electron microscopy.
Saccharomyces cerevisiae
Wild type cells of *S. cerevisiae* (BY4741) were cultured in YPD media at 30°C (11). A strain with endogenous *HSP104* C-terminally tagged with GFP (12) was used for old cell isolation, heat shock and sodium arsenite experiments. The deletion mutants *hsp104Δ* and *rpn4Δ* are from the YKO collection (EUROSCARF, Frankfurt, Germany). Strains used for investigating involvement of the ESCRT-pathway in NEB and the *pdr5Δ* strain are from this study (see yeast strains list). Transformations were performed following standard procedures (13), and gene deletions and endogenous tags were integrated via homologous recombination (14). For the hydrogen peroxide experiment, cells were grown in synthetic complete media (with yeast nitrogen base, without amino acids, pH 5.5, complete supplement mixture of amino acids and 2% glucose).
**High-pressure freezing for electron microscopy and tomography**
All samples used in this study have been prepared using high-pressure freezing followed by freeze substitution. HMC-1 cells were loaded into membrane carriers and were high-pressure frozen in a Leica EM PACT1 (Leica microsystems, Wetzlar, Germany). *T. brucei* and *S. pombe* were loaded into carriers and were high-pressure frozen in a Leica EM PACT2. *C. elegans* and *S. cerevisiae* samples were loaded into aluminum specimen carriers and were high-pressure frozen in a Wohlwend Compact 3 (M. Wohlwend GmbH, Sennwald, Switzerland). Yeast paste from wild type *S. cerevisiae* cells was used as a cryoprotectant filling the space surrounding the worms.<br>For a summary of all high-pressure freezing and freeze substitution experiments see table 1. For all samples (except *S. pombe*) a short freeze substitution protocol was applied, using 2% uranyl acetate dissolved into acetone (UA; from 20% UA stock in methanol) for one hour (2, 5, 15). To increase the penetration into intact worms, the UA solution was left on the samples for 14h (as the temperature was increased to -50ºC). The UA incubation was followed by two washes in 100% acetone for one hour

each. Before embedding, the temperature was raised from -90ºC to -50ºC overnight with a rate of 3°C/h. Samples were then embedded in K4M or HM20 resin in increasing concentrations of 20%, 40%, 50%, 80% and finally three times in 100% plastic (2 hours per solution). Polymerization of the plastic occurred over 48h using UV light at -50ºC followed by 48h in room temperature. *S. pombe* cells were high-pressure frozen and fixed through freeze substitution (long protocol) with anhydrous acetone containing 0.25% UA, 0.1% dehydrated glutaraldehyde and 0.01% osmium tetroxide (OsO_4) (7, 8, 16, 17).

For tomography, serial semi thick sections of about 210-250 nm (*S. pombe*) and 350 nm (*T. brucei* and *S. cerevisiae*) were cut and samples were poststained with 2% UA in dH_2O followed by Reyonold's lead citrate. Gold particles (15 nm) from British Bio Cell were applied to both sides of the grid to be used as fiducial markers. (4, 5, 7).

All other samples were sectioned in 70 nm thin sections and placed on either copper slot or mesh grids. Sections were stained with 2% UA for 5 minutes and Reynold's lead citrate for 1 minute (18). Washing steps after each staining were performed in dH_2O.

Immuno-electron microscopy

For the immuno-labeling experiments, the same high-pressure frozen samples embedded in HM20 resin were used, a benefit of that short FS protocol (15). For a summary of these experiments, see table 2. Grids with 70 nm thick sections were fixed in 1% paraformaldehyde (PFA) in PBS for 10 minutes. After three PBS washes of 1 minute each, samples were blocked with 0.1% fish skin gelatin and 0.8% BSA in PBS for 1 hour. For detection of NPC proteins, grids were then incubated in a 1:50 dilution of mAb414 (BioLegend, San Diego, USA) for two hours, followed by a 1:150 dilution of rabbit anti-mouse immunoglobulins (Agilent/Dako, Glostrup, Denmark) for an hour, and then a 1:70 dilution of 10 nm gold-conjugated protein A (CMC UMC Utrecht, The Netherlands) for 30 minutes. For labeling of ubiquitin, grids were incubated in a 1:20 dilution of antibody ab19247 (Abcam, Cambridge, UK) for 2 hours. Detection of GFP was performed by using a 1:5, 1:10, or 1:30 dilution of ab6556 (abcam, Camebridge, UK) and detection of Hsp104 by using a 1:100 dilution of ab69549 (abcam, Camebridge, UK) incubated overnight. Goat-anti-Rabbit IgG 10 nm gold (Electron Microscopy Sciences, Hatfield PA, USA) was then used at a 1:20 dilution for an hour. All incubations were performed at room temperature, except for the primary antibody which was kept at 4°C. Three washing steps (20 min in PBS) were carried out after incubations with each antibody. 2.5% glutaraldehyde was applied to sections for 1 hour followed by three washes (1 min in dH_2O). Sections were then contrast stained in 2% UA for 5 minutes (wash 3x 2 min in dH_2O) and 1 minute in Reynold's lead citrate (washed 5x 1 min in dH_2O).

Image acquisition and electron tomography

All thin sections were imaged at 120 kV either on a LEO 912 OMEGA (Zeiss, Krefeldt, Germany) equipped with a 2k x 2k VELETA Olympus CCD camera or on a Tecnai T12 transmission electron microscope equipped with a Ceta CMOS 16M camera (FEI Co., Eindhoven, The Netherlands). Double axis tilt series of serial sections of *T. brucei* samples were acquired every degree using the serialEM software (19) on a Tecnai TF30 300 kV IVEM microscope (FEI Co., The Netherlands) equipped with a Ultrascan 785 4k x 4k camera binned to 2k x 2k (pixel size 1 nm). For *S. pombe*, digital images (Gatan Ultrascan 890 or 895 camera, pixel size 1.5 nm) single axis tilt series were taken every 1.5° over a ±60°–65° range operating a Tecnai TF20 electron microscope (FEI Co., Eindhoven, The Netherlands) (7). For *S. cerevisiae*, double axis tilt series of serial sections were acquired on a Tecnai TF30 300 kV microscope (FEI Co., The Netherlands) equipped with a Gatan One View camera (pixel size 1.6 nm,

increment 1.5° over a ±60° range). Tomograms were acquired with the use of serialEM (19). The tomographic reconstruction was performed using of the IMOD software package (20).

Isolation of old yeast cells

Isolation of old yeast cells was performed according to Smeal et al. with some modifications in order to reduce mechanical stress and improve the cell morphology for electron microscopy (21). In brief, the cell surface of exponentially growing cells was labeled with biotin by incubating the cells with 0.5 mg/ml Sulfo-NHS-LC biotin (#21335, Thermo Fisher scientific) for 20 min at room temperature. Cells were grown in YPD, harvested prior saturation of culture, and washed in PBS + 0.5% glucose. Biotinylated cells were labeled with 17.5 ug/ml Streptavidin magnetic beads (#21344, Thermo Fisher scientific) for 1.5 hours followed by 3 x 15 min magnetic sorting with PBS + 0.5% glucose washes in between. Another two rounds of growth, streptavidin-labeling, and sorting was performed. The old cells and their unbound daughters were recovered in YPD for 4 hours before high-pressure freezing.

Stress treatments of *S. cerevisiae*

Cells were grown at 30°C to mid exponential phase. For mild heat shock, the culture was shifted to 38°C and samples were collected after 0, 5, 15, 30, 45, and 90 minutes (22). The more severe heat shock was performed at 42°C for 30 min. For hydrogen peroxide, sodium arsenite, and AZC treatments the culture was split into two, one kept as unstressed control and one treated with the stressor. Cells were exposed to 0.6 mM hydrogen peroxide for 90 minutes, 0.5 mM sodium arsenite for 60 minutes, or 1 mg/mL AZC for 30 and 90 minutes (corresponding control culture was grown for 60 minutes after mid exponential phase). Cells were harvested by filtration followed by high-pressure freezing.

Proteasome inhibition

The *rpn4Δ* strain and a wild type control was grown to mid exponential phase and prepared for high-pressure freezing. The *pdr5Δ* strain was grown to mid exponential phase prior addition of either 50 μM MG132 (#C2211, Sigma-Aldrich) dissolved in DMSO or an equivalent volume of DMSO. A 60 min incubation at 30°C followed before cells were prepared for high-pressure freezing.

Analysis of polyubiquitination

Cells were cultured and treated as described above. Mid-exponentially grown yeast corresponding to OD_{600} = 20 was harvested, washed once in distilled water and resuspended in 1 ml lysis buffer (100 mM Tris pH 7.5, 100 mM NaCl, 5 mM ethylenediaminetetraacetic acid, 1 mM dithiothreitol, 1 mM phenylmethylsulfonyl fluoride, 20 mM N-ethylmaleimide). Homogenisation was conducted via an Avestin Emulsiflex C-15, applying a homogenization pressure of 18 000 psi. Samples were cleared from cell debris and unlysed cells by centrifugation for 5 min at 3500 rcf and 4°C. 100 µl of cleared lysate was mixed with the same volume of 2x Laemmli buffer (100 mM Tris pH 6.8, 4% SDS, 20% glycerol, 0.2% bromophenol blue, 200 mM 2-mercaptoethanol) and incubated for 15 min at 95°C. The samples were then applied for SDS-PAGE and immunoblotting following standard protocols. Equal loading was controlled via Ponceau S staining directly after wet electrotransfer on PVDF membranes and blots were decorated with an anti-ubiquitin antibody (1:1000, HRP-conjugated, P4D1, sc8017, Santa Cruz). Clarity Western ECL Substrate (BIO-RAD, 1705060) and a ChemiDoc XRS+ Imaging System (BIO-RAD, 1708265) were used for detection

Analysis of cell death

Loss of membrane integrity was assessed with propidium iodide (PI) staining as previously described (23). Briefly, cells were harvested in 96-well plates after the respective stressor/mock treatment and resuspended in 250 µl of PI solution (100 µg/l PI in phosphate buffered saline PBS; 25 mM potassium phosphate, 0.9% NaCl; adjusted to pH 7.2). After 10 min incubation in the dark, cells were washed with

250 µl of PBS and analysed via flow cytometry (Guava easyCyte 5HT; Merck group). 5000 events were recorded per strain and condition using InCyte software (3.1).
**Confocal microscopy**
For visualization of nuclei, yeast cells were harvested and resuspended in DRAQ5 staining solution (5 µM DRAQ5 in PBS). After 10 min incubation in the dark, cells were washed once with PBS and immobilized on agar slides. Specimen were analyzed with a ZEISS LSM700 microscope using ZEISS ZEN blue software control. Plan-Apochromat 63x/1.40 Oil M27 objective was employed. Appropriate filter settings were used to visualize GFP and DRAQ5. Micrographs were analyzed and processed with the open-source software Fiji (24). To reduce image noise, Gaussian filtering (σ = 1) was applied, followed by background subtraction (rolling ball radius = 100 pixels). Pictures within an experiment were captured and processed in the same way.
**Phylogenetic tree**
A phylogenetic tree was constructed showing all the organisms where NEB events have been observed. The tree was generated based on the NCBI taxonomy browser for scientific names and visualized by the EvolView online software (http://www.evolgenius.info/evolview/).
**Statistics and reproducibility**
The frequency of NEB events was achieved by counting the number of events present in approximately 100-200 thin sections of nuclei. The percentage of sections containing events in each organism was presented as a bar graph. For the NPC immuno-EM assay, different cell compartments (NPCs, NEBs and lipid droplets) were categorized as labeled or not labeled based on the presence or absence of gold particles. Similarly, the percentage of labeled compartments was presented as a bar graph. For the statistical analysis, different statistical tests were examined but a non-parametric Wilcoxon test was performed as our data represent frequencies and the numerical values are not following a normal distribution nor are continuous variants (values were considered as 0 for the absence of events and 1 for the presence of events).. The test was performed using the MATLAB multi-paradigm programming language. For further details on the examined statistic tests see Supplementary table 2. For the ubiquitin and Hsp104-GFP immuno-EM, the area and number of gold particles of different cell compartments were measured using IMOD (https://bio3d.colorado.edu/imod/). The amount of gold particles per area was presented as bar graph.

1. J. H. Butterfield, D. Weiler, G. Dewald, G. J. Gleich, Establishment of an immature mast cell line from a patient with mast cell leukemia. *Leuk Res* **12**, 345-355 (1988).
2. D. Zabeo *et al.*, Exosomes purified from a single cell type have diverse morphology. *J Extracell Vesicles* **6**, 1329476 (2017).
3. H. Xiao *et al.*, Mast cell exosomes promote lung adenocarcinoma cell proliferation – role of KIT-stem cell factor signaling. *Cell Communication and Signaling* **12**, 64 (2014).
4. J. L. Höög, E. Gluenz, S. Vaughan, K. Gull, Ultrastructural investigation methods for Trypanosoma brucei. *Methods Cell Biol* **96**, 175-196 (2010).
5. J. L. Höög *et al.*, Modes of flagellar assembly in Chlamydomonas reinhardtii and Trypanosoma brucei. *eLife* **3**, e01479 (2014).
6. J. L. Höög *et al.*, 3D Architecture of the Trypanosoma brucei Flagella Connector, a Mobile Transmembrane Junction. *PLOS Neglected Tropical Diseases* **10**, e0004312 (2016).
7. J. L. Höög *et al.*, Organization of Interphase Microtubules in Fission Yeast Analyzed by Electron Tomography. *Developmental Cell* **12**, 349-361 (2007).

8. J. L. Höög, C. Antony, "Whole-Cell Investigation of Microtubule Cytoskeleton Architecture by Electron Tomography" in Methods in Cell Biology. (Academic Press, 2007), vol. 79, pp. 145-167.
9. E. Svensk *et al.*, Caenorhabditis elegans PAQR-2 and IGLR-2 Protect against Glucose Toxicity by Modulating Membrane Lipid Composition. *PLoS genetics* **12**, e1005982 (2016).
10. J. Sulston, J. Hodgkin, *Methods*, 1988 (1988).
11. Sandra M. Hill *et al.*, Asymmetric Inheritance of Aggregated Proteins and Age Reset in Yeast Are Regulated by Vac17-Dependent Vacuolar Functions. *Cell Reports* **16**, 826-838 (2016).
12. W. K. Huh *et al.*, Global analysis of protein localization in budding yeast. *Nature* **425**, 686-691 (2003).
13. R. D. Gietz, Yeast transformation by the LiAc/SS carrier DNA/PEG method. *Methods in molecular biology (Clifton, N.J.)* **1205**, 1-12 (2014).
14. C. Janke *et al.*, A versatile toolbox for PCR-based tagging of yeast genes: new fluorescent proteins, more markers and promoter substitution cassettes. *Yeast* **21**, 947-962 (2004).
15. P. Hawes, C. L. Netherton, M. Mueller, T. Wileman, P. Monaghan, Rapid freeze-substitution preserves membranes in high-pressure frozen tissue culture cells. *J Microsc* **226**, 182-189 (2007).
16. J. L. Höög *et al.*, Electron tomography reveals a flared morphology on growing microtubule ends. *Journal of Cell Science* **124**, 693-698 (2011).
17. J. L. Höög, S. M. Huisman, D. Brunner, C. Antony, Electron Tomography Reveals Novel Microtubule Lattice and Microtubule Organizing Centre Defects in +TIP Mutants. *PLOS ONE* **8**, e61698 (2013).
18. E. S. Reynolds, The use of lead citrate at high pH as an electron-opaque stain in electron microscopy. *The Journal of cell biology* **17**, 208-212 (1963).
19. D. N. Mastronarde, Automated electron microscope tomography using robust prediction of specimen movements. *Journal of Structural Biology* **152**, 36-51 (2005).
20. J. R. Kremer, D. N. Mastronarde, J. R. McIntosh, Computer Visualization of Three-Dimensional Image Data Using IMOD. *Journal of Structural Biology* **116**, 71-76 (1996).
21. T. Smeal, J. Claus, B. Kennedy, F. Cole, L. Guarente, Loss of transcriptional silencing causes sterility in old mother cells of S. cerevisiae. *Cell* **84**, 633-642 (1996).
22. K. Keuenhof *et al.*, Large Organellar Changes Occur during Mild Heat Shock in Yeast. *bioRxiv* 10.1101/2021.01.25.428102, 2021.2001.2025.428102 (2021).
23. A. Aufschnaiter *et al.*, The Enzymatic Core of the Parkinson's Disease-Associated Protein LRRK2 Impairs Mitochondrial Biogenesis in Aging Yeast. *Frontiers in Molecular Neuroscience* **11** (2018).
24. J. Schindelin *et al.*, Fiji: an open-source platform for biological-image analysis. *Nat Methods* **9**, 676-682 (2012).

Movie S1. 1.5 nm thick tomographic slices of the budding event observed in *S. pombe* (Figure 6**b**). The extra bilayer around the bud is becoming easily noticeable. The movie was created as a sequence of snap pictures acquired through the IMOD software, frame rate: 6 per second. Scale bar size is 200 nm.

Movie S2. 1.5 nm thick tomographic slices through the first budding event in the tomogram of *T. brucei* (Figure 6**e**). The movie was created as a sequence of snap pictures acquired through the IMOD software, frame rate: 6 per second. Scale bar size is 200 nm.

Movie S3. 1.5 nm thick tomographic slices through the second budding event in the tomogram of *T. brucei* (Figure 6**g**). Note the distinct electron densities at the neck of the nuclear envelope. The movie was created as a sequence of snap pictures acquired through the IMOD software, frame rate: 6 per second. Scale bar size is 200 nm.

Movie S4. 25 nm thick tomographic slices of the budding event observed in *S. cerevisiae* (Figure 4**f**). The extra bilayer around the bud is becoming easily noticeable. The movie was created as a sequence of snap pictures acquired through the IMOD software, frame rate: 6 per second. Scale bar size is 500 nm.

Movie S5. Movie of the 3D model of the budding event in *S. pombe* (Figure 6**c**). The clip was created with the use of the IMOD software, as a 360° rotation of the model. Scale bar size is 100 nm.

Movie S6. Movie of the 3D model of the first budding event in *T. brucei* (Figure 6**f**). The clip was created with the use of the IMOD software, as a 360° rotation of the model. Scale bar size is 100 nm.

Movie S7. Movie of the 3D model of the second budding event in *T. brucei* (Figure 6**h**). The clip was created with the use of the IMOD software, as a 360º rotation of the model. Scale bar size is 100 nm.

Movie S8. Movie of the 3D model of the budding event in *S. cerevisiae* (Figure 4**g**). The clip was created with the use of the IMOD software, as a 360o rotation of the model. Scale bar size is 100 nm.
